# Cultivar-specific markers, mutations, and chimerisim of Cavendish banana somaclonal variants resistant to *Fusarium oxysporum* f. sp. *cubense* tropical race 4

**DOI:** 10.1186/s12864-022-08692-5

**Published:** 2022-06-25

**Authors:** Bo-Han Hou, Yi-Heng Tsai, Ming-Hau Chiang, Shu-Ming Tsao, Shih-Hung Huang, Chih-Ping Chao, Ho-Ming Chen

**Affiliations:** 1 Agricultural Biotechnology Research Center, Academia Sinica, 11529 Taipei, Taiwan; 2 Taiwan Banana Research Institute, 90442 Pingtung, Taiwan

**Keywords:** Panama disease, RNA-seq, Single-nucleotide polymorphism, Copy number variation, Chimera

## Abstract

**Background:**

The selection of tissue culture–derived somaclonal variants of Giant Cavendish banana (*Musa* spp., Cavendish sub-group AAA) by the Taiwan Banana Research Institute (TBRI) has resulted in several cultivars resistant to *Fusarium oxysporum* f. sp. *cubense* tropical race 4 (Foc TR4), a destructive fungus threatening global banana production. However, the mutations in these somaclonal variants have not yet been determined. We performed an RNA-sequencing (RNA-seq) analysis of three TBRI Foc TR4–resistant cultivars: ‘Tai-Chiao No. 5’ (TC5), ‘Tai-Chiao No. 7’ (TC7), and ‘Formosana’ (FM), as well as their susceptible progenitor ‘Pei-Chiao’ (PC), to investigate the sequence variations among them and develop cultivar-specific markers.

**Results:**

A group of single-nucleotide variants (SNVs) specific to one cultivar were identified from the analysis of RNA-seq data and validated using Sanger sequencing from genomic DNA. Several SNVs were further converted into cleaved amplified polymorphic sequence (CAPS) markers or derived CAPS markers that could identify the three Foc TR4–resistant cultivars among 6 local and 5 international Cavendish cultivars. Compared with PC, the three resistant cultivars showed a loss or alteration of heterozygosity in some chromosomal regions, which appears to be a consequence of single-copy chromosomal deletions. Notably, TC7 and FM shared a common deletion region on chromosome 5; however, different TC7 tissues displayed varying degrees of allele ratios in this region, suggesting the presence of chimerism in TC7.

**Conclusions:**

This work demonstrates that reliable SNV markers of tissue culture–derived and propagated banana cultivars with a triploid genome can be developed through RNA-seq data analysis. Moreover, the analysis of sequence heterozygosity can uncover chromosomal deletions and chimerism in banana somaclonal variants. The markers obtained from this study will assist with the identification of TBRI Cavendish somaclonal variants for the quality control of tissue culture propagation, and the protection of breeders’ rights.

**Supplementary Information:**

The online version contains supplementary material available at 10.1186/s12864-022-08692-5.

## Background

Bananas (*Musa* spp.), perennial herbaceous crops widely grown in subtropical and tropical regions, are consumed as a staple food in many tropical countries and are a popular fruit worldwide. The vast majority of banana cultivars were derived from the inter- or intra-specific hybridization of *M. acuminata* (A genome) and *M. balbisiana* (B genome) [1, 2]. Most commercial banana cultivars are sterile and seedless because of their triploid genomes and nature of parthenocarpy [3, 4]; therefore, they are propagated vegetatively using suckers [5] or tissue culture [6, 7]. Although tissue culture facilitates the mass production of uniform and virus-free banana plantlets, the low genetic diversity of cultivated bananas makes them vulnerable to new diseases.

Fusarium wilt of banana, also known as Panama disease, is caused by the soil-borne fungal pathogen *Fusarium oxysporum* f. sp. *cubense* (Foc) [8]. The dessert banana industry based on the cultivar ‘Gros Michel’ (AAA genome) was destroyed by the Foc strain designated as race 1 (R1). Because of their good resistance to Foc R1, cultivars in the Cavendish banana subgroup (AAA genome) replaced ‘Gros Michel’ in the 1950s and have dominated the global banana market since then. However, Cavendish cultivars are susceptible to the Foc strain designated as tropical race 4 (TR4) [8, 9], which is virulent and aggressive. Foc TR4 has spread around the world and is threatening global banana production [10–13]. In 2020, two isolates of Foc R1 were reported to be able to infect Cavendish bananas [14]. The effective control of Foc using fungicides is not available [15]; thus, the best strategy to combat Foc TR4 is to identify or generate resistant cultivars of commercial value.

The improvement of Cavendish banana resistance to Foc TR4 has been conducted through transgenic or mutagenesis approaches because the triploid and sterile nature of commercial banana cultivars limits the success of conventional breeding. The expression of a resistance gene encoding a nucleotide-binding leucine-rich repeat protein from a wild diploid subspecies of *M. acuminata* with resistance to Foc TR4 increased the Foc TR4 resistance of the Cavendish cultivar ‘Grand Nain’ in field trials [16]. Mutagenesis of the Cavendish banana has been successfully established by treating shoot apical meristems with chemical mutagens or gamma irradiation [17, 18]. Similar to the somaclonal variation observed in the tissue culture of other crops [19], morphological and DNA variations were reported in tissue culture–derived Cavendish banana plants [20]. Although most tissue culture–derived somaclonal variants are associated with undesirable traits, breeders have selected some somaclonal variations to successfully improve various traits of many crops, including Foc TR4 resistance in the Cavendish banana [7, 21, 22]. Through a large-scale screening of plantlets derived from the tissue culture of a Foc TR4–susceptible Cavendish cultivar, ‘Pei-Chiao’ (PC), in fields heavily infested with Foc TR4 over many years, the Taiwan Banana Research Institute (TBRI) recovered dozens of somaclonal variants with varying degrees of resistance to Foc TR4, which were named the Giant Cavendish Tissue Culture Variants (GCTCVs) [21]. The further selection of these somaclonal variants for superior horticultural traits in yield, growth rate, and eating quality resulted in several Foc TR4–resistant cultivars of commercial value, such as the moderately resistant ‘Tai-Chiao No. 5’ (TC5) and the highly resistant ‘Formosana’ (FM) (also known as GCTCV-218) and ‘Tai-Chiao No. 7’ (TC7) lines [22, 23]. Owing to its stable Foc TR4 resistance and good horticultural traits, FM has become a popular option used by banana growers to combat Foc TR4 in the Philippines and Mozambique [24].

Tissue culture can induce genetic and epigenetic changes in regenerated plants [25–27]; for example, base substitutions, small-scale deletions or insertions, and transpositions of transposons have been observed in rice (*Oryza sativa*) regenerants [28]. Regenerants also frequently have abnormal chromosome numbers or chromosomal aberrations, including duplications, deletions, and unbalanced translocations [29, 30]. DNA methylation changes have also been reported in regenerants of a wide range of plant species and implicated in the phenotypic variations caused by tissue culture [26, 31, 32]. Genetic and epigenetic changes in plant regenerants from tissue culture have been investigated using cytogenetic, polymerase chain reaction (PCR), and enzyme digestion approaches [25, 26, 29, 30, 33], but recent advances in microarray and next-generation sequencing technologies are now enabling the genome-wide exploration of these genetic and epigenetic changes [34, 35].

The tissue culture–derived Foc TR4–resistant cultivars of Giant Cavendish bananas selected by TBRI are valuable resources for sustaining banana production under the threat of this devastating fungal disease; however, the genetic or genomic differences between these somaclonal variants and their susceptible progenitor have not yet been investigated. A lack of sequence information has retarded the development of molecular markers for these Foc TR4–resistant cultivars and the exploration of underlying disease resistance mechanisms. Through the analysis of sequence variations in RNA-sequencing (RNA-seq) data, we successfully identified single-nucleotide variants (SNVs) specific to TC5, TC7, and FM and converted several SNVs into cleaved amplified polymorphic sequence (CAPS) markers or derived-CAPS (dCAPS) markers. An analysis of sequence variations also revealed chromosomal deletions in these three cultivars and chimerism in TC7. These findings demonstrate the effective use of RNA-seq data in the detection of sequence and structural variations in triploid bananas, which are relevant to the identification and further characterization of these banana somaclonal variants.

## Results

### Discovery of cultivar-specific SNVs from RNA-seq data

The development of single-nucleotide polymorphism (SNP) markers from expressed sequence tags or RNA-seq data has been demonstrated in various crops, such as pineapple (*Ananas comosus*) [36], radish (*Raphanus raphanistrum* subsp. *sativus*) [37], and barley (*Hordeum vulgare*) [38]. Given the continuous propagation of commercial banana plantlets through tissue culture, new mutations are very likely to be introduced into the individual clones of progenitor cultivars, as well as their somaclonal variants [39]. This could increase heterogeneity among individual clones within cultivars and pose a critical challenge for marker development for commercial banana cultivars.

**Fig. 1:**
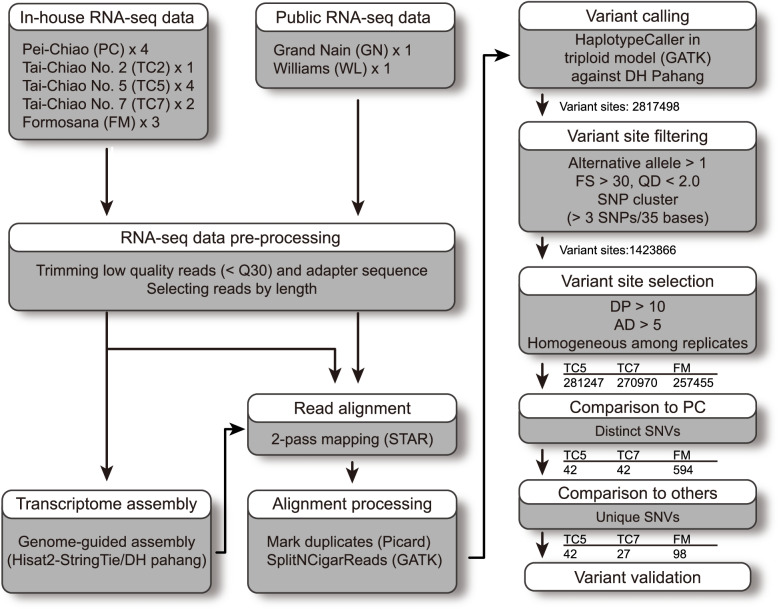
Pipeline for identifying unique SNVs in three TBRI Foc TR4–resistant cultivars using RNA-seq data. The numbers of SNV sites retained after each step are indicated. SplitNCigarReads, split reads with N in Cigar; DP, depth coverage; AD, depth of coverage of each allele per sample; QD, variant confidence normalized by the unfiltered depth of variant samples; FS, strand bias estimated using Fisher’s exact test

To overcome this challenge, we performed an RNA-seq analysis of four samples of PC and TC5, three samples of FM, and two samples of TC7. Including replicates for each cultivar could reduce the detection of false cultivar-specific markers caused by SNVs in individual clones or sequencing errors. Notably, the RNA-seq analysis of PC, TC5, and TC7 included the replicates that were harvested and sequenced in different batches and years (Table S1). This would further benefit the identification of cultivar-specific markers that are stable over generations. In addition to PC, we also sequenced another Foc TR4–susceptible cultivar, ‘Tai-Chiao No. 2’ (TC2). TC2 was not derived from the Giant Cavendish PC, which was introduced from China mainland, but a Cavendish banana from a farm in Barbados. Taking advantage of Cavendish banana RNA-seq data in the public domain, the sequences of two popular commercial banana cultivars, ‘Grand Nain’ and ‘Williams’, were also included in our analysis [40, 41]. We established a pipeline containing multiple stringent filtering and selection steps to identify cultivar-specific SNVs of triploid bananas from RNA-seq data (Fig. 1). The numbers of raw reads in the 16 RNA-seq libraries we analyzed ranged from 21,589,694 to 67,353,186 (Table S1). The shorter raw reads of‘Grand Nain’ and ‘Williams’ decreased the number of mapped reads. After read preprocessing and genome-guided transcript assembly using the reference genome sequence of double haploid (DH) ‘Pahang’ banana [42], we obtained sequences for 33,475 loci in the Cavendish bananas we analyzed.

By comparing with the DH ‘Pahang’ sequences, sequence variants were identified in each sample of the seven cultivars using GATK HaplotypeCaller [43] with a haploid setting of three. We uncovered 2,817,498 sites harboring at least one variant in one of the 16 samples (Fig. 1), which were further filtered by allele type, alternative allele number, SNP cluster, strand bias, and variant confidence. The filtering narrowed down the number of variant sites to 1,423,866. Next, we selected variant sites that had good coverage for each sample and each allele to reduce the false variants caused by sequencing errors or random fluctuations of sequencing reads. Variant sites that exhibited distinct alleles among the replicates within the cultivars were removed because they might represent mutations in individual clones. After the selection, 281,247, 270,970, and 257,455 variant sites in TC5, TC7, and FM, respectively, were subjected to further analysis. In the comparison with the PC sequences, 42, 42, and 594 sites had distinct alleles in TC5, TC7, and FM, respectively (Fig. 1). After the removal of SNVs that were shared between any two of the seven cultivars, we obtained 42, 27, and 98 unique SNVs for TC5, TC7, and FM, respectively. Some of these SNVs were subjected to further validation using Sanger sequencing from genomic DNA isolated from samples independent of those used for the RNA-seq analysis.

### Sequence heterozygosity in the Giant Cavendish banana transcriptome

**Fig. 2:**
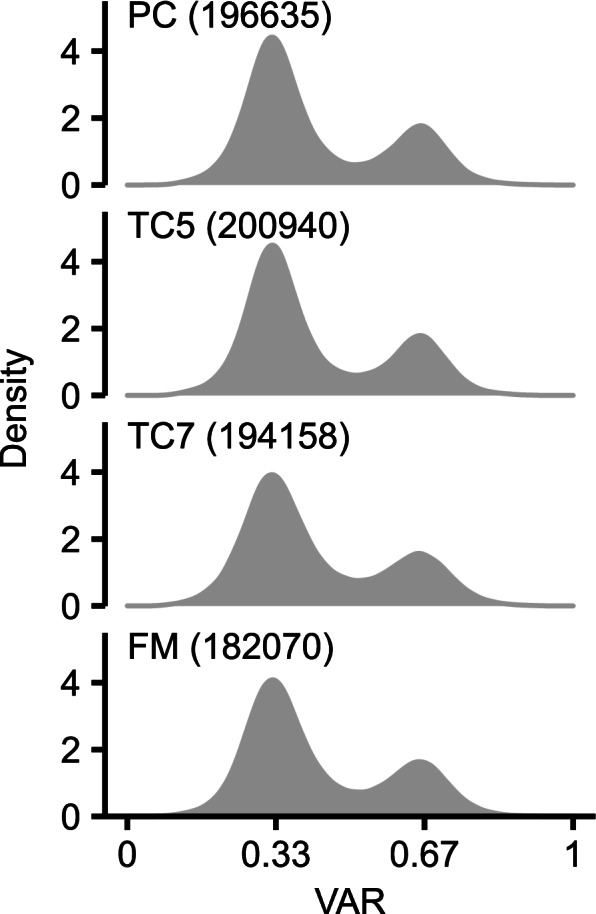
Density distribution of the variant allele ratio (VAR) of TBRI banana RNA-seq data at heterozygous sites. The VAR was calculated as the read count of the variant allele divided by the total read count at each heterozygous site. The number of heterozygous sites included in each plot is indicated in parentheses

Previous analyses using whole-genome sequencing, genotyping-by-sequencing, and RNA-seq revealed a substantial degree of sequence heterozygosity in various diploid and triploid banana species and subspecies [44–46]. Notably, a previous analysis of heterozygous sites recovered from Dwarf Cavendish banana whole-genome sequencing data revealed two major peaks in the distribution of the reference allele ratio (the sequence count matching a reference allele divided by the total count at that position) [44]; one peaked at around 0.33 (i.e., one copy of the reference allele and two copies of the alternative allele) and the other peaked at around 0.67 (i.e., two copies of the reference allele and one copy of the alternative allele). Similarly, we also observed two major peaks at around 0.33 and 0.67 when analyzing the variant allele ratio (VAR) of a large number of heterozygous sites (> 180,000) identified from the RNA-seq data of four TBRI cultivars (Fig. 2). Moreover, consistent with the previous finding from the Dwarf Cavendish banana [44], the VAR peak around 0.33 was twice as high as that around 0.67. The result suggested that the pipeline we built for the analysis of sequence variations in RNA-seq data could faithfully capture DNA sequence variations in triploid banana genomes to some extent.

### A small SNV hotspot on TC5 chromosome 4

**Fig. 3:**
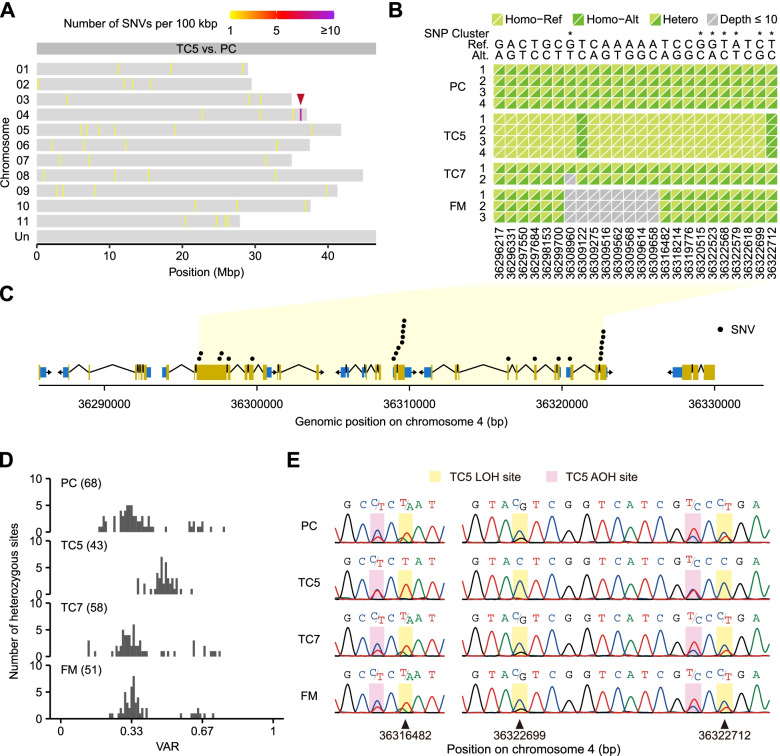
A SNV-rich region on TC5 chromosome 4. **A** Genomic distribution of distinct SNVs between TC5 and PC. A red arrowhead indicates a region enriched with distinct SNVs. **B** Alleles at the SNV sites in the TC5 SNV-rich region. For each diallelic site, reference (Ref) or alternative (Alt) alleles in a homozygous (homo) or heterozygous (hetero) state are shown as a color composite. Asterisks mark the sites within SNP clusters. **C** Gene models in the TC5 SNV-rich region. Blue boxes indicate UTRs, yellow boxes indicate CDSs, and thin lines indicate introns. **D** Distribution of the variant allele ratio (VAR) at heterozygous sites in the TC5 SNV-rich region. The VAR was calculated as the read count of the variant allele divided by the total read count at each heterozygous site. The number of heterozygous sites analyzed in each plot is indicated in parentheses. **E** Selective validation of loss of heterozygosity (LOH) and alteration of heterozygosity (AOH) in the TC5 SNV-rich region using Sanger sequencing from genomic DNA

A total of 42 distinct SNVs between TC5 and PC were dispersed among the 11 banana chromosomes, with a hotspot in a region spanning coordinates 36,296,217–36,322,712 on chromosome 4 (Fig. 3A). At all of the 24 SNV sites (including seven located in SNP clusters defined by GATK) in this 26-kbp region, four PC samples were uniformly heterozygous for the reference and alternative alleles (Fig. 3B). By contrast, samples of TC5 but not TC7 or FM showed a loss of heterozygosity (LOH) at all of these 24 sites, of which 22 sites exclusively contained the reference allele. Given that Cavendish bananas are triploid, sterile, and propagated vegetatively, the uniform LOH was more likely attributed to a single-copy deletion of this region rather than dense point mutations in the six genes spanning this region (Fig. 3C). In addition to the LOH, we inferred that a single-copy deletion in the triploid genome would also change the ratio of two alleles (i.e., from 2:1 or 1:2 to 1:1) at the sites that remained heterozygous in this region. Indeed, the VAR distribution in this region of TC5 was different from that of the other three cultivars (Fig. 3D), for which the VAR distribution peaked at around 0.33, similar to that of the whole transcriptome (Fig. 2); by contrast, TC5 had fewer heterozygous sites and the VAR distribution peaked at around 0.5. The LOH and change of VAR that caused the alteration of heterozygosity (AOH) at selected sites in this region were confirmed using Sanger sequencing from genomic DNA (Fig. 3E). Taken together, these results indicated that TC5 lost a copy of a small segment of chromosome 4, leading to a copy number change of six genes. The results also demonstrated the potential of using RNA-seq data to detect copy number variations in triploid banana genomes through the analysis of sequence heterozygosity.

### A large SNV-rich region on FM chromosome 5

**Fig. 4:**
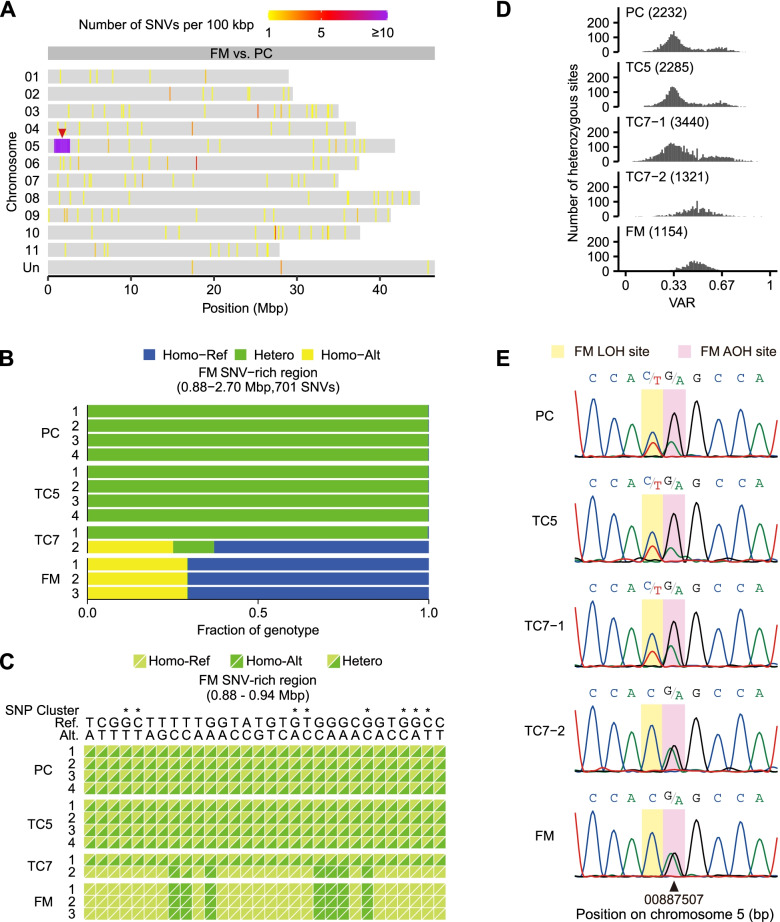
A SNV-rich region on FM chromosome 5. **A** Genomic distribution of distinct SNVs between FM and PC. A red arrowhead indicates the region most enriched with distinct SNVs. **B** Composition of alleles at the SNV sites in the FM SNV-rich region. SNV sites were classified as homozygous (homo) for reference (Ref) or alterative (Alt) alleles or heterozygous (hetero) for two alleles. **C** Alleles at the selected SNV sites in the FM SNV-rich region. For each diallelic site, reference or alternative alleles in a homozygous or heterozygous state are shown as a color composite. Asterisks mark the sites within SNP clusters. **D** Distribution of the variant allele ratio (VAR) at heterozygous sites in the FM SNV-rich region. The VAR was calculated as the read count of the variant allele divided by the total read count at each heterozygous site. The number of heterozygous sites analyzed in each plot is indicated in parentheses. **E** Selective validation of loss of heterozygosity (LOH) and alteration of heterozygosity (AOH) in the FM SNV-rich region using Sanger sequencing from genomic DNA

Among the 594 distinct SNVs between FM and PC, 492 were located in a region spanning coordinates 0.88–2.70 Mbp of chromosome 5, while the others were dispersed across the genome (Fig. 4A). Similar to the SNV hotspot on TC5 chromosome 4, nearly all distinct SNVs in this region (including those in SNV clusters) were homozygous for either the reference or alternative alleles in FM but heterozygous in all the PC and TC5 samples (Fig. 4B C). Intriguingly, the two TC7 samples harbored distinct genotypes at these sites: Most were heterozygous in TC7-1, while about 88% were homozygous in TC7-2. The LOH suggests a single-copy loss of this chromosome 5 region in FM. This speculation was further supported by a reduced number of heterozygous sites and a shift of the major VAR peak from 0.33 to 0.5 in FM (Fig. 4D). Sanger sequencing from genomic DNA confirms the LOH and AOH at selected sites in this region (Fig. 4E). Again, the results imply that FM lost a copy of this 1.82-Mbp segment of chromosome 5, which might affect the expression level or protein polymorphism of the 213 genes within.

### LOH and AOH in multiple regions of TC7 chromosome 5

**Fig. 5:**
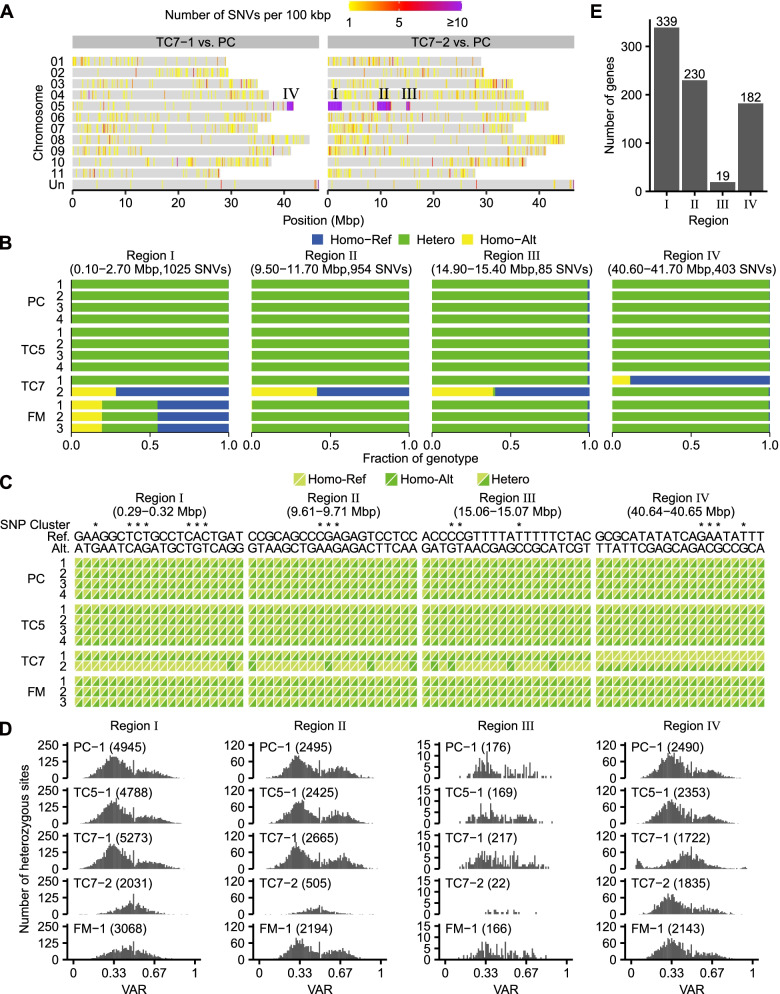
Change of heterozygosity in multiple regions of TC7 chromosome 5. **A** Genomic distribution of distinct SNVs between TC7 individual samples and the PC group. Roman numerals indicate SNV-rich regions. **B** Composition of alleles at SNV sites in the four indicated TC7 SNV-rich regions. SNV sites were classified as homozygous (homo) for reference (Ref) or alterative (Alt) alleles or heterozygous (hetero) for two alleles. **C** Alleles at selected SNV sites in the four TC7 SNV-rich regions. For each diallelic site, reference or alternative alleles in a homozygous or heterozygous state are shown as a color composite. Asterisks mark the sites falling within SNP clusters. **D** Distribution of the variant allele ratio (VAR) at the heterozygous sites in the four TC7 SNV-rich regions. The VAR was calculated as the read count of the variant allele divided by the total read count at each heterozygous site. The numbers of heterozygous sites analyzed in each plot are indicated in parentheses. **E** Numbers of genes in the four TC7 SNV-rich regions

The SNVs that were homogenous among replicates within cultivars but distinct between TC7 and PC were not highly enriched in any particular genomic region (Fig. S1). Nevertheless, the analysis of sequences differing between the individual TC7 samples and the PC group revealed evident but different SNV-rich regions on chromosome 5 (Fig. 5A). TC7-1 had a SNV-rich region at the 3′ terminus (region IV), whereas TC7-2 had three SNV-rich regions of various sizes on the 5′ arm (regions I, II, and III). This type of difference was not observed among the four TC5 or three FM individual samples (Fig. S2). The sizes of these four SNV-rich regions ranged from 0.5 to 2.6 Mbp and contained 85 to 1025 distinct SNVs (Fig. 5B). Remarkably, region I in TC7-2 overlapped the SNV-rich region in FM (Figs. 4 and 5A). Similar to the SNV hotspots on TC5 chromosome 4 and FM chromosome 5, these four SNV-rich regions on TC7 chromosome 5 also showed a LOH, resulting in homozygous reference or alternative alleles (Fig. 5B C). In addition, compared with the individual samples of PC and TC5, the two TC7 samples had fewer heterozygous sites and a distinct VAR distribution in these regions (Fig. 5D). The dramatic change of sequence heterozygosity suggests a single-copy deletion of these chromosome 5 regions, which might reduce the expression level or protein polymorphism of the 19 to 339 genes they each contain (Fig. 5E). The different SNV hotspots found in the two TC7 samples suggests that these structural variants on chromosome 5 might be heterogeneous among individual TC7 clones or the cells within one individual. To know the incidences of these two types, we genotyped roots of 54 TC7 plantlets. The result confirmed the occurrences of both types discovered from the RNA-seq analysis but suggested that the deletions on chromosome 5 in TC7-2 are more common among TC7 plantlets, as 50 out of the 54 roots we genotyped showed a LOH in region I, while only four roots showed a LOH in region IV (Fig. S3). It remains unknown whether both TC7 types are resistant to Foc TR4.

### Chimerism in TC7 clones

**Fig. 6:**
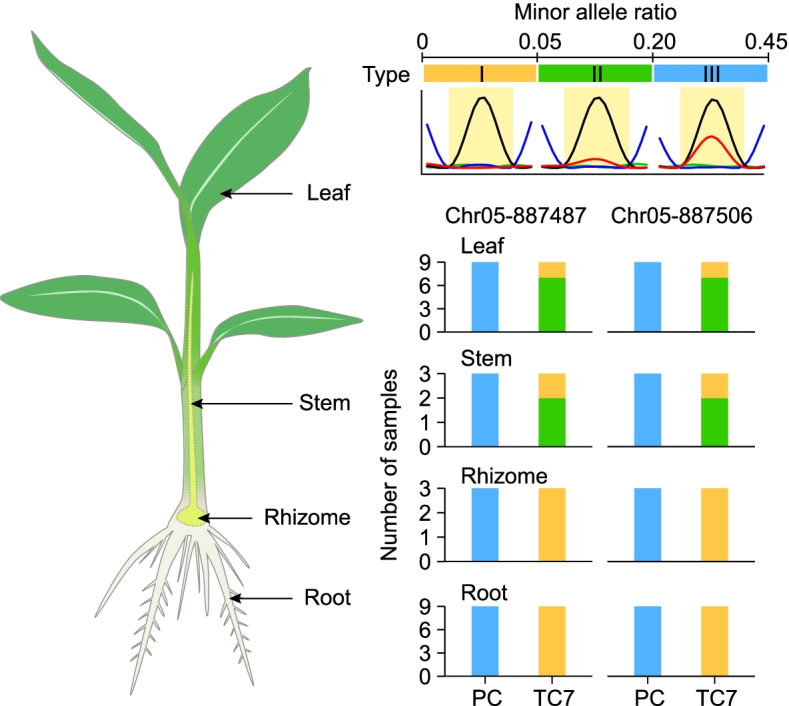
Distinct genotypes among the tissues of TC7 plants. Four types of tissues from three soil-grown PC and TC7 individuals were genotyped at two sites in TC7 SNV-rich region I on chromosome 5 using Sanger sequencing from genomic DNA. For each plant, three leaves, one stem, one rhizome, and three root samples were genotyped. The minor allele ratio (mAR) was calculated as the ratio of the height of the minor allele peak to the total height of the major and minor allele peaks at the site in the Sanger sequencing chromatogram. Based on the mAR, the genotypes were classified into three categories: type I (mAR < 0.05), type II (0.05 ≤ mAR < 0.20), and type III (0.2 ≤ mAR < 0.45)

Somatic mutations occurring in tissue-cultured plants might not be uniformly distributed in entire plants, which might instead be chimeric, meaning somaclonal variants containing mixed cells of different genotypes. The different SNV-rich regions detected in two TC7 samples imply that the TC7 plants giving rise to the samples we used for RNA-seq analysis could be chimeras of more than one genotype for these regions on chromosome 5. To test this possibility, we genotyped four tissues (leaf, stem, rhizome, and root) from three soil-grown PC and TC7 individuals using Sanger sequencing from genomic DNA. For each plant, we genotyped three leaves, one stem, one rhizome, and three roots, specifically focusing on two variant sites in the SNV-rich region I on chromosome 5 where TC7-2 showed a LOH. Based on the peaks in the sequencing chromatograms, we then calculated the minor allele ratio (mAR) as the ratio of the height of the minor allele to the total height of the major and minor alleles at each site. All PC samples for the four tissues exhibited a similar degree of heterozygosity, with a mAR ≥ 0.2 (type III) (Fig. 6). By contrast, the minor allele was barely detected (mAR < 0.05, type I) at both sites in the nine root samples and three rhizome samples from three TC7 plants (Fig. 6), confirming the LOH observed in the TC7-2 RNA-seq data (Fig. 5B). This result suggests that the cells of these two tissues from the assayed TC7 plantlets likely all possessed a genome with a single-copy deletion in the genotyping region. Although the minor allele was detected in seven leaf and two stem samples of TC7, it was underrepresented (0.05 ≤ mAR < 0.2, type II) compared with any tissue of PC (mAR ≥ 0.2, type III) (Fig. 6). Notably, similar to the root and rhizome samples, one stem and two leaf samples from one TC7 plant had no signal for the minor allele at either site. A decrease rather than a complete loss of the minor allele in most leaf and stem samples implies that a few cells in these two TC7 tissues might retain three copies of this genotyping region; therefore, distinct genotypes among TC7 tissues and an underrepresented level of the minor allele together support chimerism in TC7, especially in the leaves and stems.

### Validation of RNA-derived SNV markers using Sanger sequencing

**Fig. 7:**
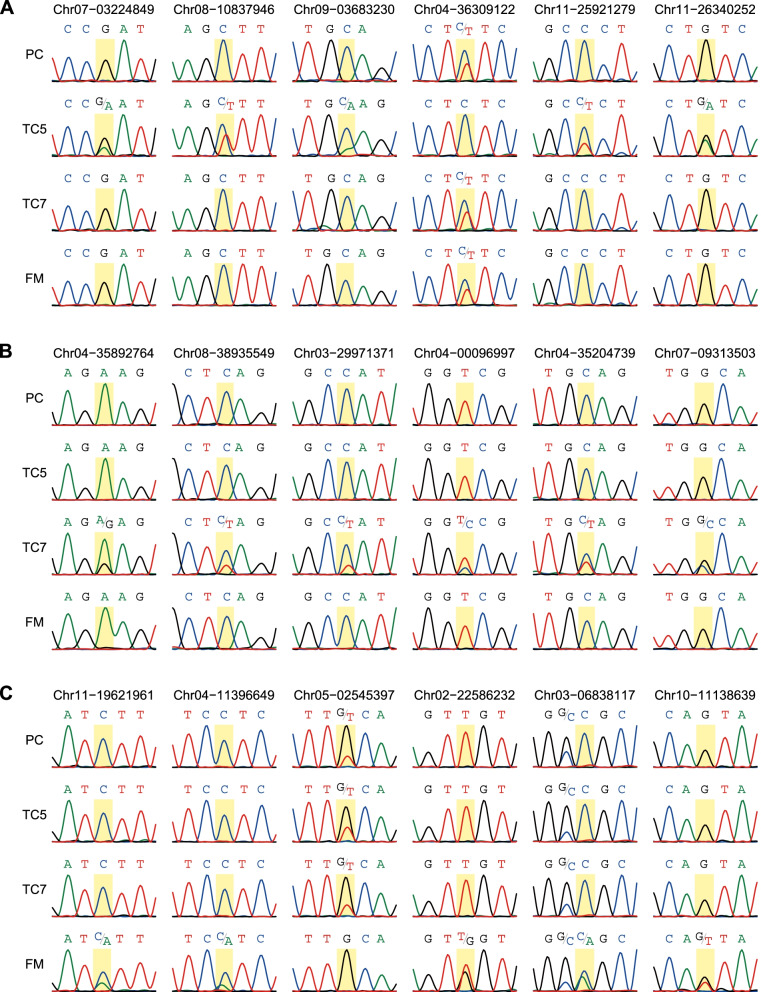
Validation of TC5-, TC7-, and FM-specific SNV markers obtained from the RNA-seq data using Sanger sequencing from genomic DNA. **A**–**C** Sequencing chromatograms of the four cultivars at the sites harboring SNVs specific to TC5 (**A**), TC7 (**B**), and FM (**C**)

To develop reliable SNV markers for the three TBRI Foc TR4–resistant cultivars, we selected some cultivar-specific SNVs identified from the RNA-seq analysis for further validation using Sanger sequencing. Based on the sequence counts, 11, 10, and 8 SNVs were selected for TC5, TC7, and FM, respectively, for validation with the DNA isolated from samples that differed from those used for the RNA-seq analysis. With the exception of the four selected SNVs for TC7, the selected SNVs were successfully detected using the primers we designed and showed distinct alleles between the targeted cultivar and others in the sequencing chromatograms (Fig. 7 and S4). Most of the validated cultivar-specific SNVs harbored heterozygous alleles in the targeted cultivar but homozygous alleles in others. Furthermore, the chromatogram height of the allele shared among the four cultivars was often 1.5- to 3-fold higher than that of the allele specific to one cultivar, implying a mutation in one copy of the three homologous chromosomes (Fig. 7). The lower validation rate of TC7-specific SNVs might be due to the facts that only two TC7 samples were used in the RNA-seq analysis and that this line appears to be chimeric. This result implies that increasing replicate numbers may help to eliminate false markers caused by sequence variation among individuals.

### Development of CAPS/dCAPS markers for TBRI cultivar identification

**Fig. 8:**
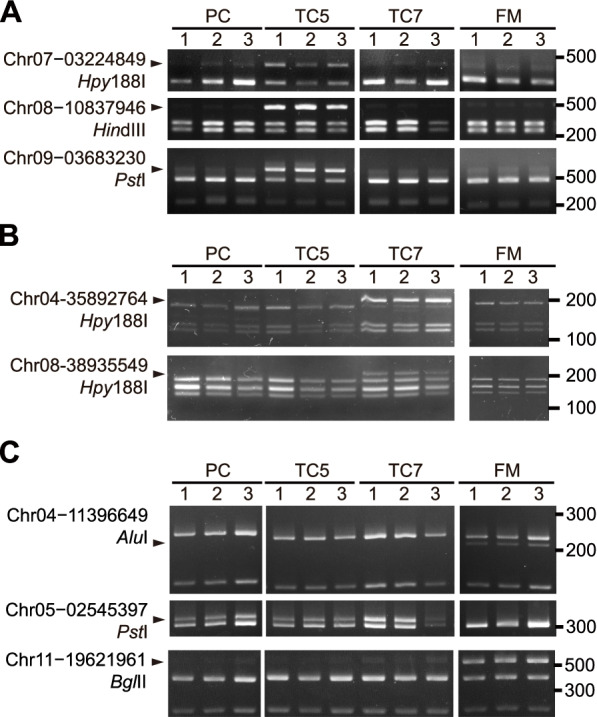
Validation of SNV-derived CAPS/dCAPS markers of TC5, TC7, and FM. **A–C** Electrophoresis profiles of CAPS/dCAPS products for the TC5 (**A**), TC7 (**B**), and FM (**C**) markers. The SNV sites converted into CAPS/dCAPS markers and the restriction enzymes used are shown on the left of the electrophoresis profiles. The numbers above each profile indicate independent replicates of a cultivar. An arrowhead to the left indicates the cultivar-specific product of the enzyme digestion, and a number to the right of the profile shows the marker size in base pairs. The uncropped full-length gels are presented in Figs. S5, S6, and S7

To apply cultivar-specific SNVs to the identification of TBRI cultivars without sequencing, we converted several Sanger sequencing–validated SNVs into CAPS or dCAPS markers and tested their specificity with multiple samples for each cultivar. Both CAPS and dCAPS are non-sequencing approaches for SNV detection, which involve PCR, restriction enzyme digestion, and gel electrophoresis [47]. Unlike the CAPS assays that detect SNVs embedded in restriction enzyme recognition sites, dCAPS assays create restriction enzyme recognition sites in PCR products at the target SNV sites using primers that have mismatches to the DNA template. For TC5, we successfully developed three CAPS markers which detected the SNVs on chromosomes 7, 8, and 9 through digestion with *Hpy*188I, *Hin*dIII, and *Pst*I, respectively (Figs. 7 and 8A). At these three SNV sites, TC5 exhibited heterozygous alleles, while the others had homozygous alleles. Only the PCR product derived from the gene copy harboring the cultivar-specific allele, but not from the other two copies, was resistant to enzyme digestion. As expected, compared with the samples of the other three cultivars, the TC5 samples all showed an extra band of digestion products with an expected size of 455 to 623 bp (Fig. 8A and Table S2). For TC7, two SNVs on chromosomes 4 and 8 were converted into dCAPS markers through the digestion of PCR products using *Hpy*188I (Fig. 8B). Heterozygous alleles were only identified in TC7 and were expected to result in an extra band of digestion products with a size of about 200 bp (Fig. 7B and Table S2). Indeed, only the TC7 samples harbored an extra band of the expected size (Fig. 8B). For FM, three SNVs on chromosomes 4, 5, and 11 were converted into dCAPS or CAPS markers through digestion with *Alu*I, *Pst*I, and *Bgl*II, respectively (Fig. 8C). Similar to the CAPS/dCAPS markers of TC5 and TC7 (Fig. 8A and 8B), only FM samples showed an extra band of the FM CAPS marker on chromosome 11 and the dCAPS marker on chromosome 4, as these SNV sites were heterozygous in FM but homozygous in the others (Figs. 7 and 8C). On the contrary, the FM-specific SNV on chromosome 5 was homozygous in FM but heterozygous in the other genotypes (Fig. 7C), with the other three cultivars producing an extra band of this dCAPS marker (Fig. 8C). The result of the CAPS/dCAPS assays with multiple samples for each cultivar again confirmed that the selected SNVs are common and specific to the plantlets of the targeted cultivars. As the samples we used for the CAPS/dCAPS assays and the RNA-seq analysis were derived from the plants cultivated in different years, the results thus also suggested that the SNV markers we identified are stable over generations.

### Specificity of the CAPS/dCAPS markers among Cavendish banana cultivars

**Fig. 9:**
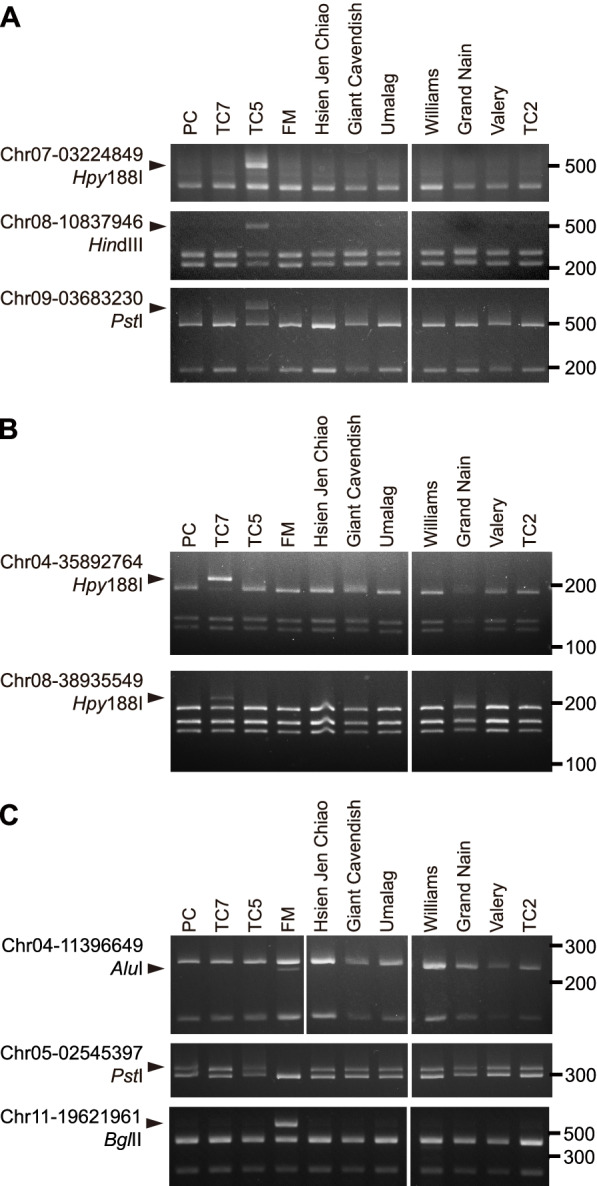
Specificity of TC5, TC7, and FM CAPS/dCAPS markers among 11 Cavendish banana cultivars. **A–C** Electrophoresis profiles of CAPS/dCAPS products for the TC5 (**A**), TC7 (**B**), and FM (**C**) markers. The SNV sites converted into CAPS/dCAPS markers and the restriction enzymes used are shown on the left of the electrophoresis profiles. An arrowhead to the left indicates the cultivar-specific product of the enzyme digestion, and numbers to the right of the profile show the marker sizes in base pairs. The uncropped full-length gels are presented in Figs. S8, S9, and S10

We then further examined the specificity of these CAPS/dCAPS markers with two additional TBRI cultivars, ‘Hsien Jen Chiao’ and TC2 (Table S3). ‘Hsien Jen Chiao’ is also a somaclonal variant of PC but with little Foc TR4 resistance. In addition, we also tested five important commercial cultivars, which are Foc TR4–susceptible, ‘Giant Cavendish’ and ‘Umalag’ from the Philippines, ‘Williams’ from Hawaii, USA, and ‘Grand Nain’ and ‘Valery’ from Honduras. The specific digestion products of the CAPS/dCAPS assays were only detected or absent in the targeted TBRI cultivars (Fig. 9). This result demonstrates the high specificity of these CAPS/dCAPS markers in identifying these three TBRI cultivars among PC-related or distantly related Cavendish cultivars.

## Discussion

Somatic mutation is particularly important for the improvement of triploid bananas as they have limited ability to incorporate new genetic variation through sexual reproduction. Through a transcriptomic sequence variation analysis (Fig. 1), we identified unique SNVs and chromosomal deletions in three tissue culture–derived somaclonal variants of Giant Cavendish banana that are resistant to Foc TR4 (Figs. 1, 3, 4 and 5, and 7). Two of these Foc TR4–resistant somaclonal variants, TC7 and FM, share a common single-copy deletion region on chromosome 5. The CAPS/dCAPS markers converted from the SNVs can specifically identify these somaclonal variants among local and international Cavendish cultivars (Figs. 8 and 9). Using these markers, TBRI can detect these somaclonal variants from specific DNA changes in TC5, TC7 and FM, and confirm that tissue culture approach via somaclonal mutation can be used for variety improvement. We also revealed that one of the somaclonal variants, TC7, appears to be a chimera, as its genotype of the deletion regions on chromosome 5 varied among tissues (Fig. 6). Our work demonstrates that with proper design and advanced analysis, profiling the transcriptome can reveal mutations including structural variations in tissue culture–derived and –propagated bananas.

### Heterozygosity of banana genomes

Since most bananas are hybrids both between and within species and subspecies [1, 2], their genomes are presumed to be heterozygous. Indeed, the analysis of SNP, insertion, and deletion markers in 105 diploid banana accessions by Sardos et al. revealed an average heterozygosity rate of 0.19 for the wild and seeded accessions and 0.29 for the cultivated and unseeded accessions [46]. For commercial cultivars of Cavendish banana, which has a triploid genome and is sterile, the genetic loci of heterozygous alleles are not able to become homozygous through sexual reproduction. Moreover, regeneration through tissue culture enhances the occurrence of mutations, which has been demonstrated in model organisms as well as crops [25, 48–50]. Along with an increase in the number of subcultures, the number of somatic mutations within and among individual clones is also increased, which is likely associated with the elevated production of off-types. Tissue culture–induced base substitutions in homozygous loci increase the heterozygosity of cultivated bananas. Consistent with a previous analysis of the Dwarf Cavendish genome assembly [44], the transcriptomic sequence variation analysis performed in this study also recovered a large number of loci with heterozygous alleles (Fig. 2). Although a high degree of heterozygosity may complicate the assembly of the three haplophases of the Cavendish genome, our results demonstrate that the analysis of heterozygosity could give insights into the copy number variation in triploid banana genomes (Figs. 3 and 4, and 5). A similar approach has been used in the detection of copy number variations in cancer cells and somaclonal variants of grapevine (*Vitis vinifera*) [51, 52]. With modifications to fit a model for triploid genomes, the tools developed previously to detect copy number variations in the analysis of heterozygosity might be applicable to banana RNA-seq data; however, the boundaries of duplication or deletion regions might not be delineated accurately by the heterozygosity analysis as some genes lack sequence variations.

### Chromosomal deletions in vegetatively propagated crops

Compared with other vegetatively propagated crops, such as grapevine and potato (*Solanum tuberosum*), which remain able to reproduce sexually, the bananas of a triploid genome must be propagated clonally, which abolishes the possibility of eliminating chromosomes with large deletions or extra chromosomal segments through meiosis. Moreover, the transposon activation and instability of DNA methylation induced by tissue culture may enhance the occurrence of chromosome breakage [53]; therefore, large chromosomal deletions might be more common in tissue culture–propagated banana cultivars. Our identification of one or multiple chromosomal deletions in these three TBRI somaclonal variants supports this scenario (Figs. 3 and 4, and 5). A single-copy deletion in a triploid banana genome might have a profound effect if only the deleted copy is functional. Alternatively, if three copies of a gene have a dosage effect or produce polymorphic protein products because of sequence variations, a single-copy deletion might also lead to a new trait. Previously, heterozygous chromosomal deletions removing the functional genes involved in anthocyanin biosynthesis were shown to account for the white-skinned and grey-skinned grapes of ‘Pinot blanc’ and ‘Pinot gris’, respectively [54, 55]. Notably, the deletions on chromosome 5 of TC7-2 and FM overlap, making this region an attractive candidate locus for Foc TR4 resistance. The overlapping region might contain one or several susceptibility genes that assist Foc TR4 invasion, proliferation or systemic spread or act as negative regulators of plant immunity. Hence, the loss of this region could enhance the Cavendish banana resistance to Foc TR4.

### Chimeras in somaclonal variants

Somatic mutations occurring spontaneously or induced during tissue culture will only exist in single cells at first. The cell lineage derived from the mutated cell may spread as a sector or eventually dominate the entire plant through plant growth or regeneration. Depending on the composition of cells with and without somatic mutations in the meristem, chimeras can be divided into two major categories, periclinal and sectorial [56]. For periclinal chimeras, cells with a somatic mutation completely occupy a specific cell layer but are absent from other cell layers. In sectorial chimeras, all cell layers in the meristem contain some cells carrying the somatic mutation. Genotyping various TC7 tissues yielded a result (Fig. 6) similar to that of the grapevine somaclonal variant ‘Pinot gris’, which forms grey-skinned fruits. This cultivar is a periclinal chimera in which the inner layer (L2) cells have a large deletion on chromosome 2, which accounts for the color change of the grapes [54]. This layer-specific chromosomal deletion in ‘Pinot gris’ was supported by genotyping differences between tissues comprising both the outer cell layer (L1) and the L2 layer, and those comprising solely L2 cells. In the chromosomal deletion region, tissues from the progenitor cultivar with purple grapes, ‘Pinot noir’, all harbored heterozygous alleles with a ratio close to 1:1; however, in ‘Pinot gris’, the pure L2-derived tissues (the root and fruit flesh) exhibited a LOH, whereas the tissues derived from both layers (the leaf and fruit skin) exhibited heterozygous alleles but with a ratio deviated from 1:1 [54]. A layer-specific chromosomal deletion might have also occurred in TC7. All of the TC7 root and rhizome samples we examined uniformly showed a LOH, suggesting that all L2 cells have lost the copy carrying the minor allele (Fig. 6). By contrast, compared with PC tissues, most TC7 leaf and stem samples possessed a very weak signal for the minor allele, implying that some cells in the L1 layer might retain the copy carrying the minor allele.

### Development of molecular markers for somaclonal variants

Experimental designs and analytic pipelines are important for the success of developing molecular markers for somaclonal variants. As highlighted by Michno and Stupar in the analysis of genomic variation in transgenic soybean [57], genomic heterogeneity was present between individuals of the progenitor lines for transformation and regeneration. Although bananas are propagated through tissue cultures, individuals derived from a cultivar might still be heterogeneous. In addition, chimerism caused by tissue culturing might introduce heterogeneity between the cells of an individual and further complicate marker development for banana cultivars, as was the case for TC7 (Figs. 5 and 6). Increasing the number of biological replicates of each cultivar could reduce the false markers arising from genetic heterogeneity or sequencing errors. Filtering SNVs with a low coverage is also crucial for removing unreliable SNVs. With sufficient biological replicates and stringent filtering criteria, molecular markers for somaclonal variants can be successfully developed from RNA-seq data; however, the reliable SNVs identified from the transcriptome would be enriched for more highly expressed genes. The selection of somaclonal variants has led to new cultivars of assorted crop species other than banana [7]. Some of them, such as potato, grapevine, strawberry (*Fragaria × ananassa*), and sugarcane (*Saccharum* sp.), are also propagated clonally. The successful development of molecular markers from RNA-seq data for the verification of three somaclonal variants of Cavendish banana in this study suggests the potential to apply similar approaches to somaclonal variants of other species.

## Conclusions

In addition to the identification of differentially expressed genes, as demonstrated in this study, RNA-seq data can be applied to the development of reliable molecular markers and the detection of chromosomal deletions for triploid and tissue culture–derived banana cultivars. The SNV markers developed in this study have good specificity and reproducibility to distinguish TBRI Cavendish somaclonal variants of very high genetic similarity.

## Methods

### Plant materials

The Cavendish bananas (*Musa* spp., AAA group) used for RNA-seq or genotyping in this study were obtained from the Taiwan Banana Research Institute (TBRI, Pingtung, Taiwan). Shoots of unrooted or rooted plantlets (1–2 months) in tissue culture jars were harvested for RNA-seq analysis. Details of the samples used in RNA-seq and the 11 samples used in the CAPS/dCAPS marker analysis are included in Tables S1 and S3, respectively.

### RNA sequencing

Total RNA was extracted using PureLink Plant RNA reagent (Thermo Fisher Scientific). The RNA-seq libraries were prepared from the extracted RNA using either the NEBNext Ultra RNA Library Prep Kit for Illumina (New England Biolabs) or the TruSeq RNA Library Prep Kit v. 2 (Illumina), following the manufacturers’ instructions. Paired-end sequencing was performed on an Illumina HiSeq 2500 or 4000, or a NovaSeq 6000, following the standard protocols, to produce reads of 126 or 150 nt (Table S1).

### Analysis of sequence variations from RNA-seq data

RNA-seq data were generated for five TBRI cultivars (PC, TC2, TC5, TC7, and FM) in this study, while RNA-seq data of ‘Grand Nain’ and ‘Williams’ were downloaded from the NCBI database (accession numbers are listed in Table S1). After trimming the adapter sequences and low-quality bases with a Phred quality score less than 30 using Trimmomatic v. 0.39 [58], the RNA-seq reads shorter than 90 nt (for ‘Williams’) or 100 nt (for the other genotypes) were discarded. The remaining reads from the five TBRI cultivars that were aligned to the banana genome of *M. acuminata* DH ‘Pahang’ (v. 2; http://banana-genome-hub.southgreen.fr) using HISAT2 v. 2.1.0 [59] and StringTie v. 2.0.4 were included in the genome-guided transcriptome assembly [60]. The assembled transcripts of the five cultivars and the annotated transcripts of DH ‘Pahang’ were compared and merged using GffCompare v. 0.11.5 [61]. For variant calling, the trimmed reads were mapped to the DH ‘Pahang’ genome with transcriptome assembly data using STAR v. 2.7.0f in two-pass mapping mode [62]. Next, reads with multiple genomic hits or marked as potential PCR duplicates by Picard v. 2.21.2 in GATK v. 4.1.4.1 [43] were removed. SplitNCigarReads in GATK v. 4.1.4.1 was used to hard clip the sequences of alignment sticking out over introns and reassign mapping qualities for uniquely mapped reads. After this post-processing read alignment, the variants were called using GATK haplotype caller in a triploid model and with a minimum Phred-scaled confidence threshold of 20. The SNV sites that were located in a cluster of more than three SNVs in a 35-bp region, with more than one alternative allele, a Fisher Strand value larger than 30, or a Qual By Depth value smaller than 2.0 were filtered out. The variants that passed the filtering were further selected using a custom Perl script if they were homogeneous among the replicates within cultivars and had a total depth of coverage higher than 10 and depth of coverage for each allele greater than 5. For the resulting SNVs, analyses of VAR at heterozygous sites and comparisons between cultivars were performed using custom R scripts.

### DNA isolation

For genotyping using Sanger sequencing or CAPS/dCAPS analyses, the banana tissues were ground into a fine powder in liquid nitrogen, and about 0.1 mg of powder was used for the DNA isolation. The DNA of various tissues was extracted using tris-phosphate ethylenediaminetetraacetic acid buffer (100 mM Tris-HCl (pH 9.5), 1 M KCl, 10 mM EDTA (pH 8.0)), following the protocol described previously [63] or using a DNeasy plant mini kit (Qiagen) according to the instructions.

### Genotyping by Sanger sequencing

To confirm the SNVs identified from the RNA-seq data, PCR primers were designed using Primer3 (v. 4.1.0; http://primer3.ut.ee/) [64] to amplify the sequences upstream and downstream of the SNV for use in Sanger sequencing. The sequences of the primers are listed in Table S4. Each 20-µl PCR reaction contained 10 µl of 2× master mix buffer (TE-SR01, TOOLS), 1 µl of 10 µM each of the forward and reverse primers, 0.2–1.0 µl of genomic DNA (1–10 ng µl^-1^) and 7–7.8 µl of ultrapure water. For all of the primer pairs listed in Table S4, the PCR program started with a 1-min denaturation at 95 °C; followed by 30–35 cycles of 30 s denaturation at 95 °C, 30 s annealing at 55 °C, and 30 s elongation at 72 °C; and ended with a 5-min extension at 72 °C. PCR products of expected sizes were sequenced by the Sanger method.

### CAPS and dCAPS analyses

Cultivar-specific SNVs confirmed using Sanger sequencing were selected for the development of CAPS or dCAPS markers. The primers used for the validation of TC5-specific SNVs using Sanger sequencing were also employed in the CAPS analysis. The dCAPS primers, which introduced a restriction enzyme site to the amplification products of the sequences shared among four cultivars but not the sequence specific to one cultivar, were designed using dCAPS Finder 2.0 (http://helix.wustl.edu/dcaps/dcaps.html) [65]. The primer sequences for the CAPS and dCAPS analyses are listed in Table S2. The PCR mixture and program for the CAPS and dCAPS analyses were the same as for genotyping by Sanger sequencing. A 15-µl aliquot of unpurified PCR products were digested for 2 h at 37 °C using 30 units of the appropriate restriction enzymes given in Table S2. After digestion, 10 µl of PCR product was analyzed on 1–5% agarose gels according to the expected sizes of the digestion products.

## Supplementary Information

**Additional file 1: Figure S1-S10** and **Table S1-S4**.

## Data Availability

The RNA-seq data generated in this study are available in the NCBI Sequence Read Archive (http://www.ncbi.nlm.nih.gov/sra) under accession number PRJNA658209.

## Abbreviations

AOH: Alteration of heterozygosity
CAPS: Cleaved amplified polymorphic sequence
dCAPS: Derived cleaved amplified polymorphic sequence
DH: Double haploid
FM: ‘Formosana’
Foc: *Fusarium oxysporum* f. sp. *cubense*
LOH: Loss of heterozygosity
mAR: Minor allele ratio
PC: ‘Pei-Chiao’
PCR: Polymerase chain reaction
SNP: Single-nucleotide polymorphism
SNV: Single-nucleotide variant
TBRI: Taiwan Banana Research Institute
TC5: ‘Tai-Chiao No. 5’
TC7: ‘Tai-Chiao No. 7’
TR4: Tropical race 4
VAR: Variant allele ratio
