# Supplementary material for: Cultivar-specific markers, mutations, and chimerisim of Cavendish banana somaclonal variants resistant to Fusarium oxysporum f. sp. cubense tropical race 4
Source: BMC Genomics. 2022 Jun 25;23:470. doi: 10.1186/s12864-022-08692-5 (PMC9233791; doi:10.1186/s12864-022-08692-5)
Supplement: Supplementary file 1 — Additional file 1: Figure S1-S10 and Table S1-S4. [file 12864_2022_8692_MOESM1_ESM.pdf]

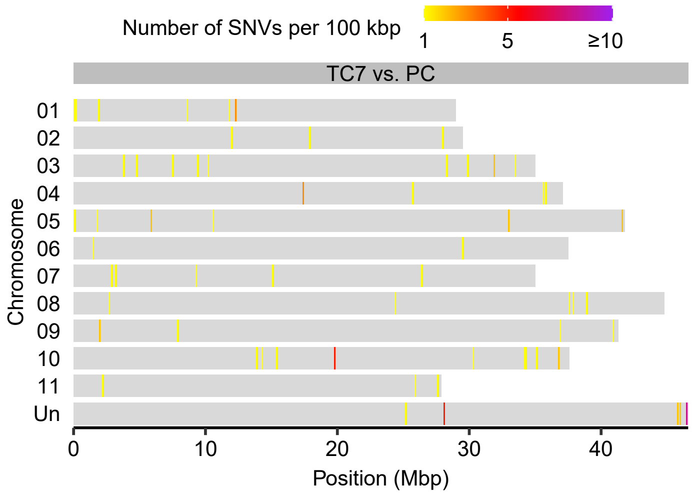

Figure S1. Genomic distribution of distinct SNVs between the TC7 and PC groups.

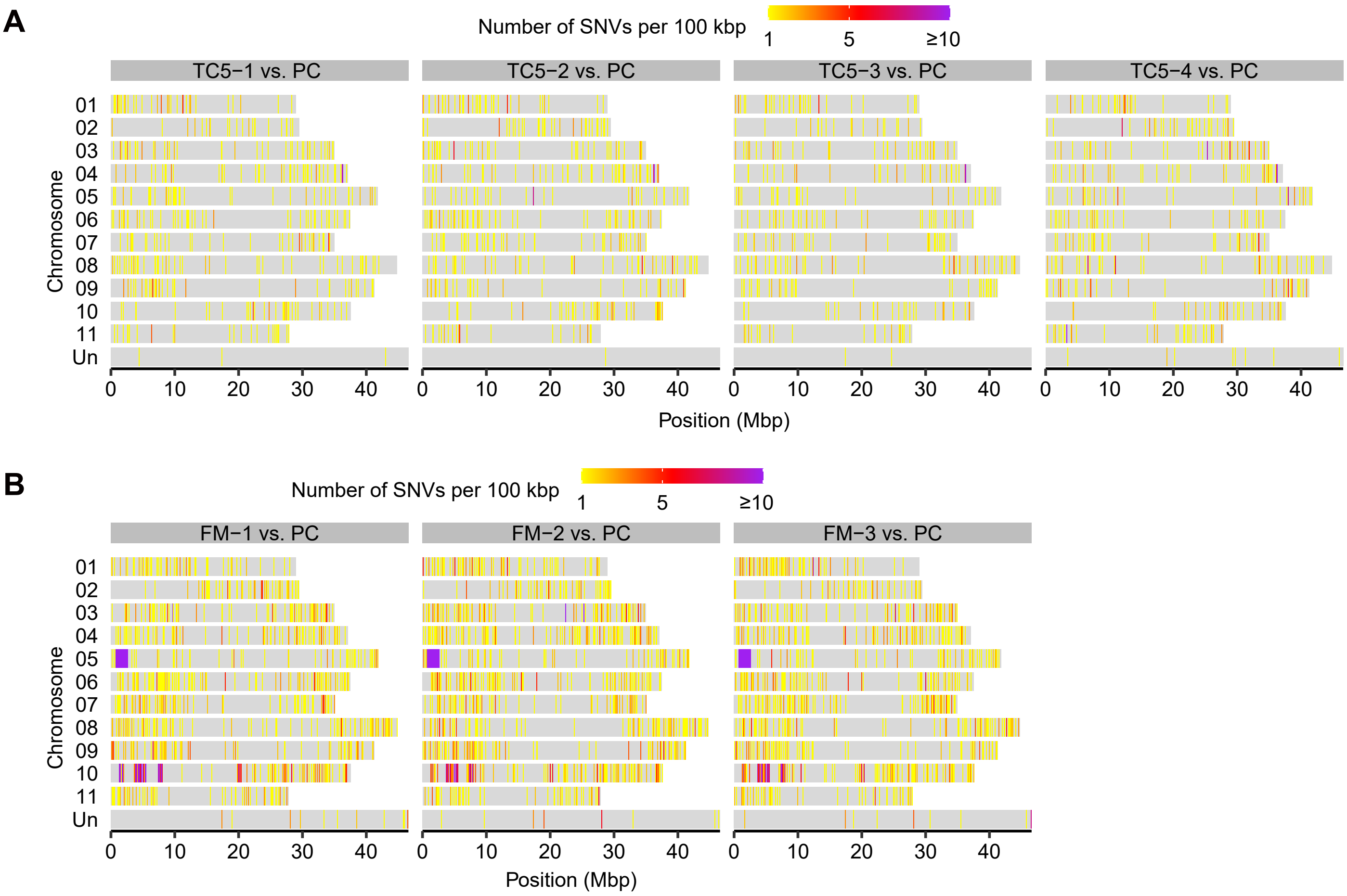

Figure S2. Genomic distribution of distinct SNVs between the individual samples of two Foc TR4–resistant cultivars and the PC group. **(A)** Distinct SNVs between the individual TC5 samples and the PC group. **(B)** Distinct SNVs between the individual FM samples and the PC group.

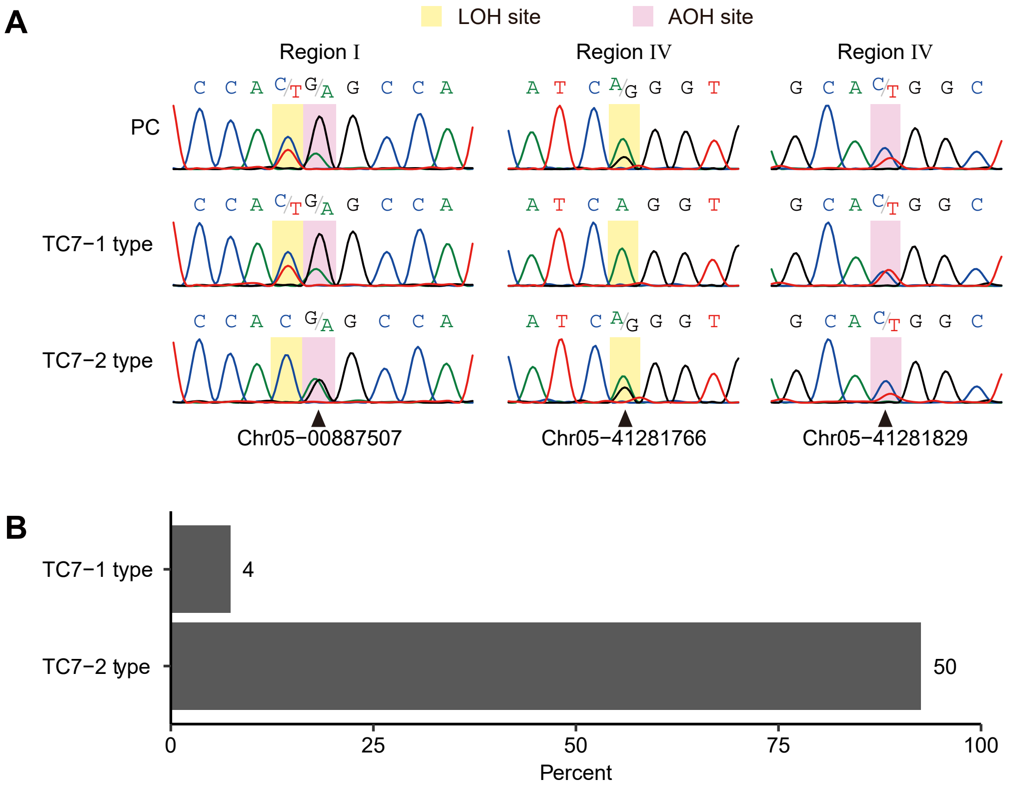

Figure S3. Predominance of TC7 roots with LOH and AOH in chromosome 5 region I. **(A)** Representative sequencing chromatograms of TC7 roots showing LOH and AOH in region IV (TC7-1 type) or region I (TC7-2 type) **(B)** Genotype counts in 54 TC7 roots.

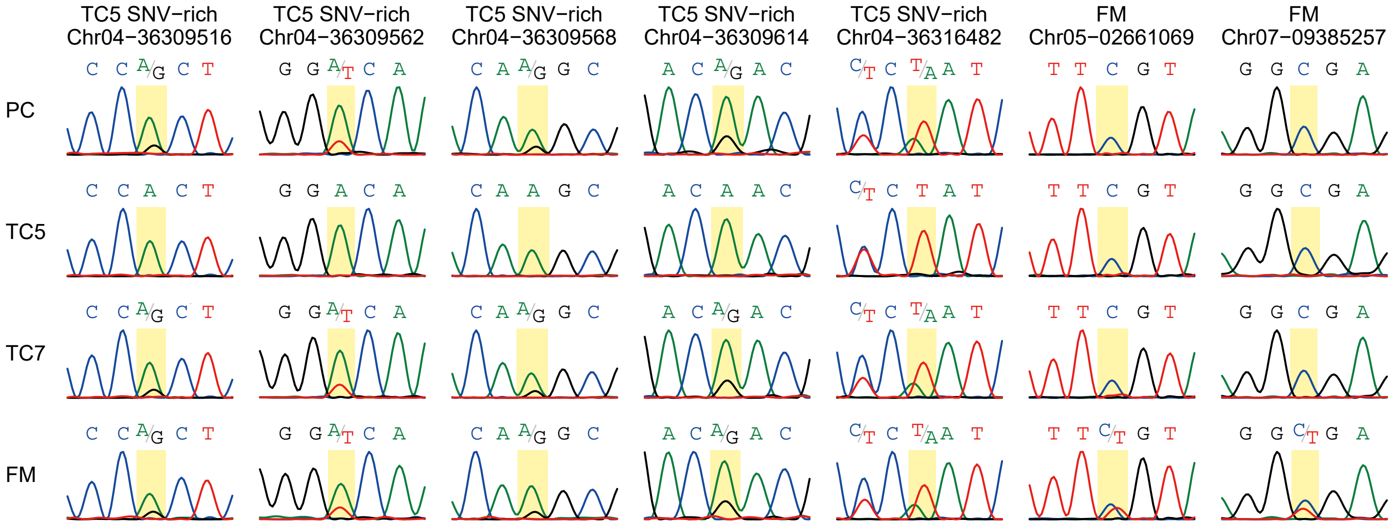

Figure S4. Five TC5-specific and two FM-specific SNVs obtained from the RNA-seq data analysis, validated using Sanger sequencing from genomic DNA.

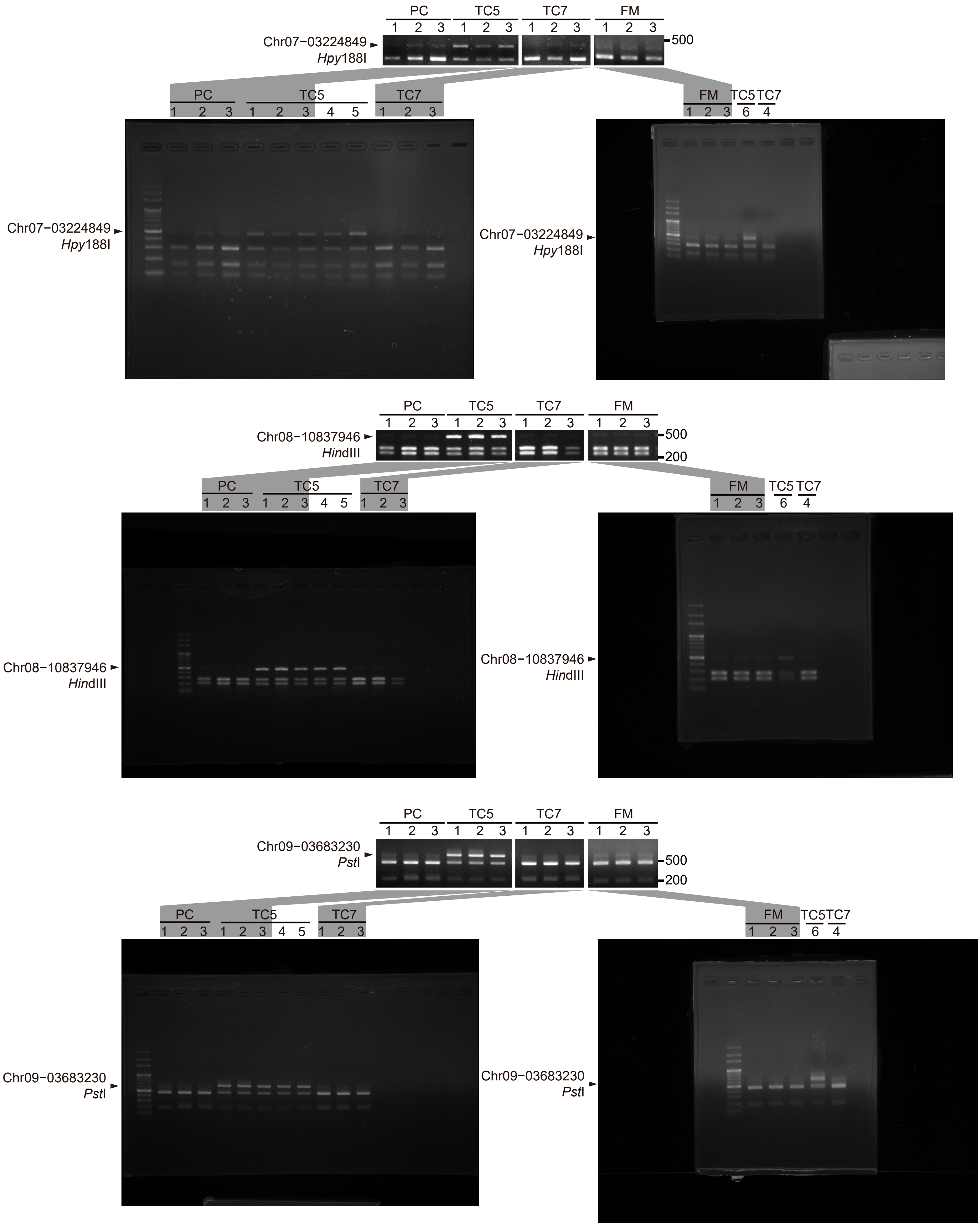

Figure S5. The uncropped full-length gels for TC5 CAPS/dCAPS marker in Figure 8A.

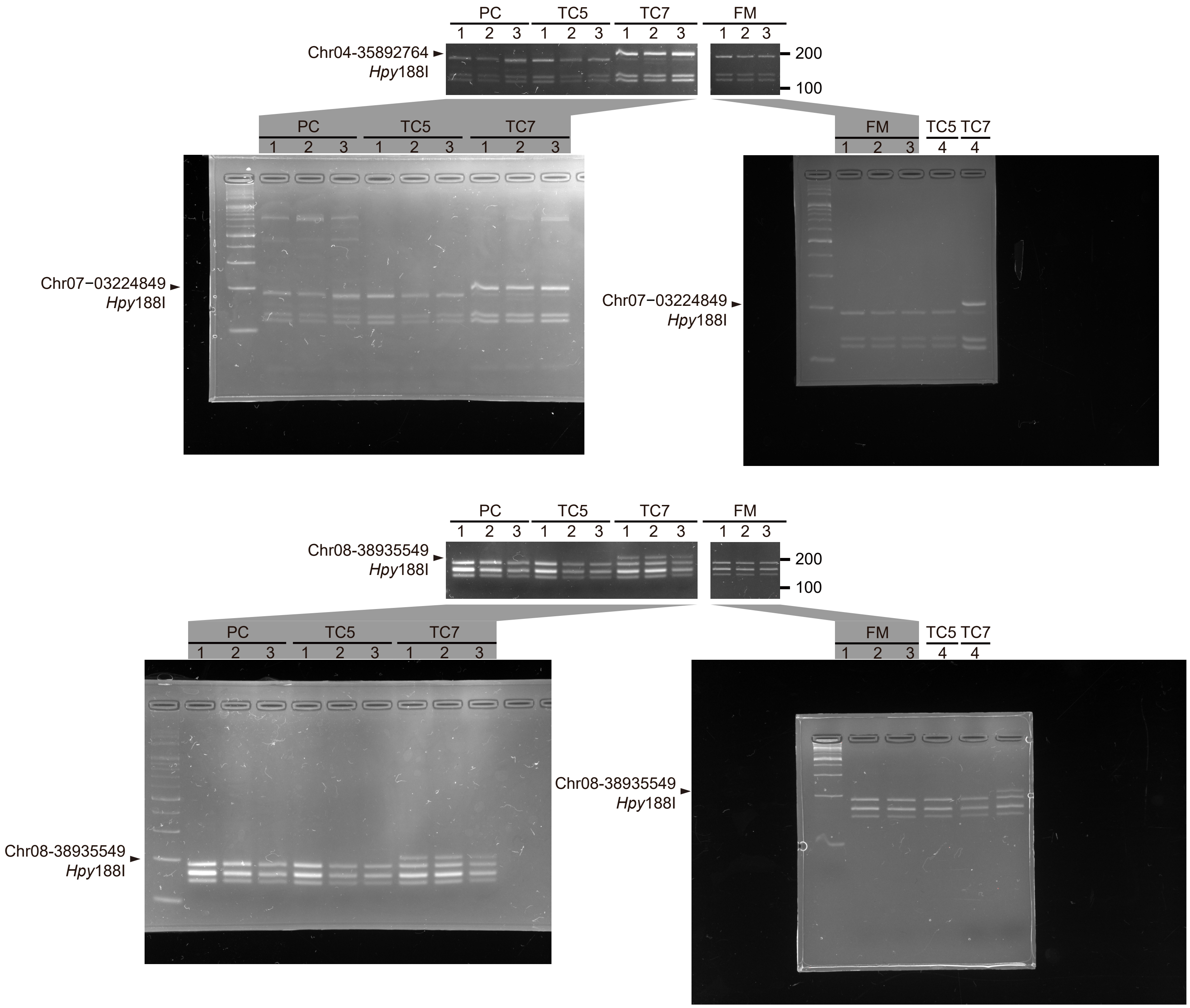

Figure S6. The uncropped full-length gels for TC7 CAPS/dCAPS marker in Figure 8B.

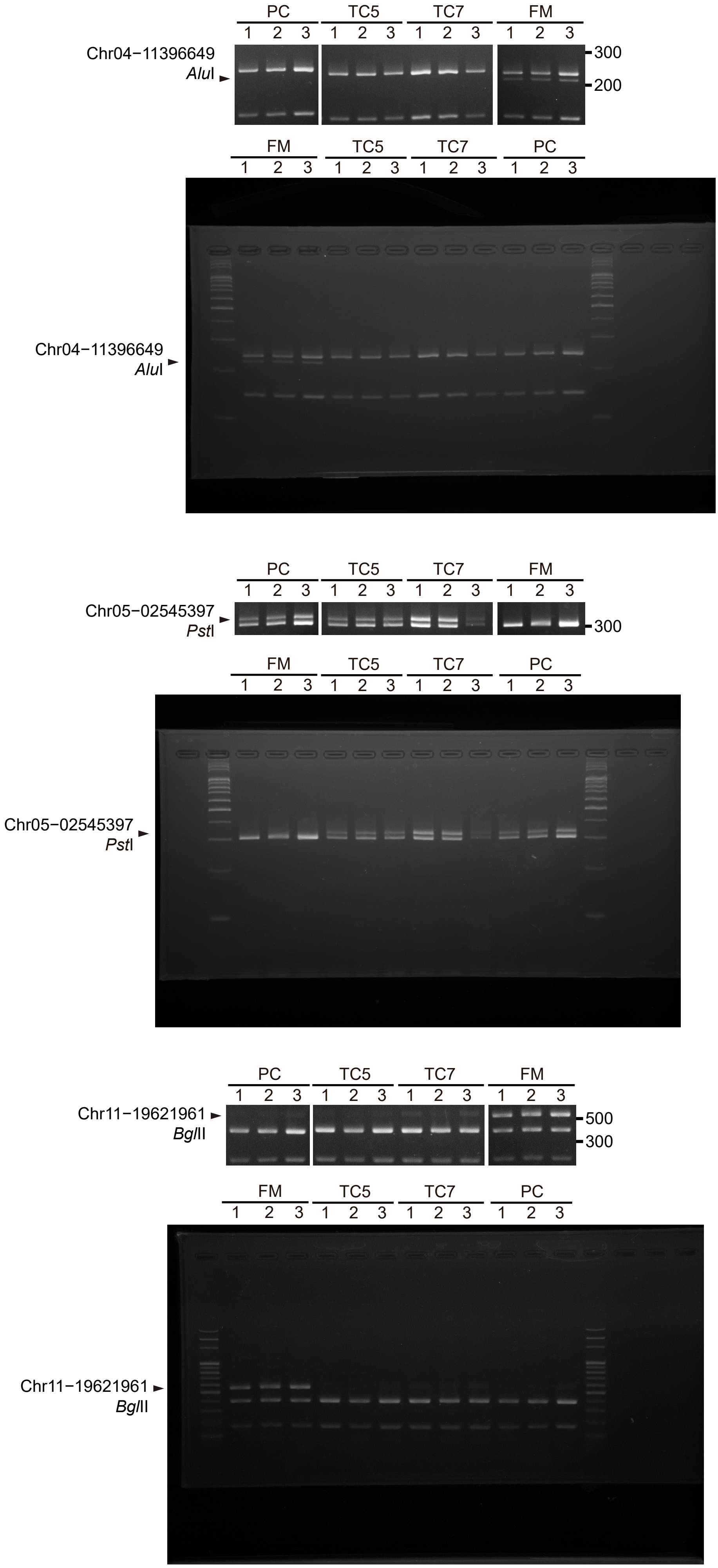

Figure S7. The uncropped full-length gels for FM CAPS/dCAPS marker in Figure 8C.

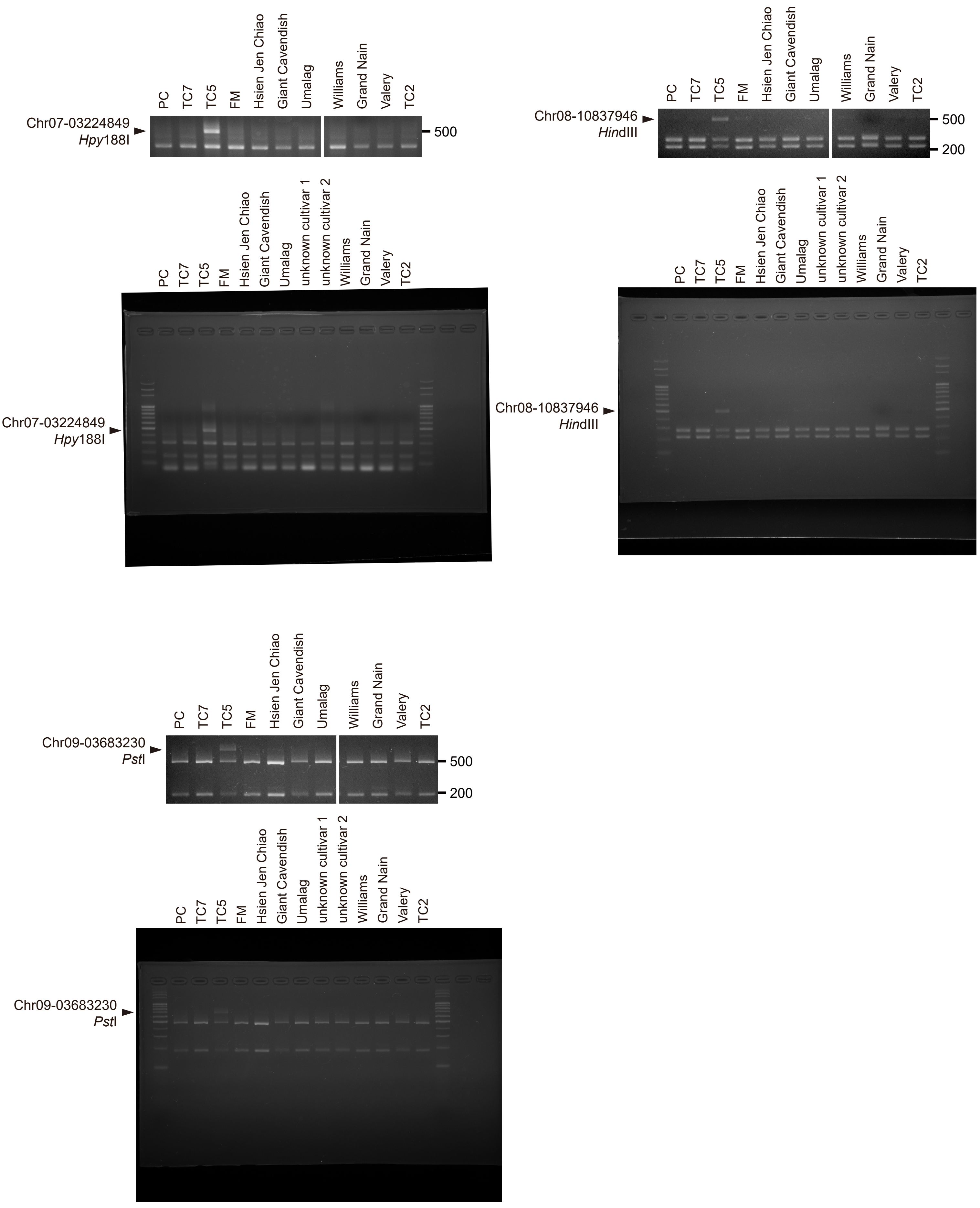

Figure S8. The uncropped full-length gels for TC5 CAPS/dCAPS marker in Figure 9A.

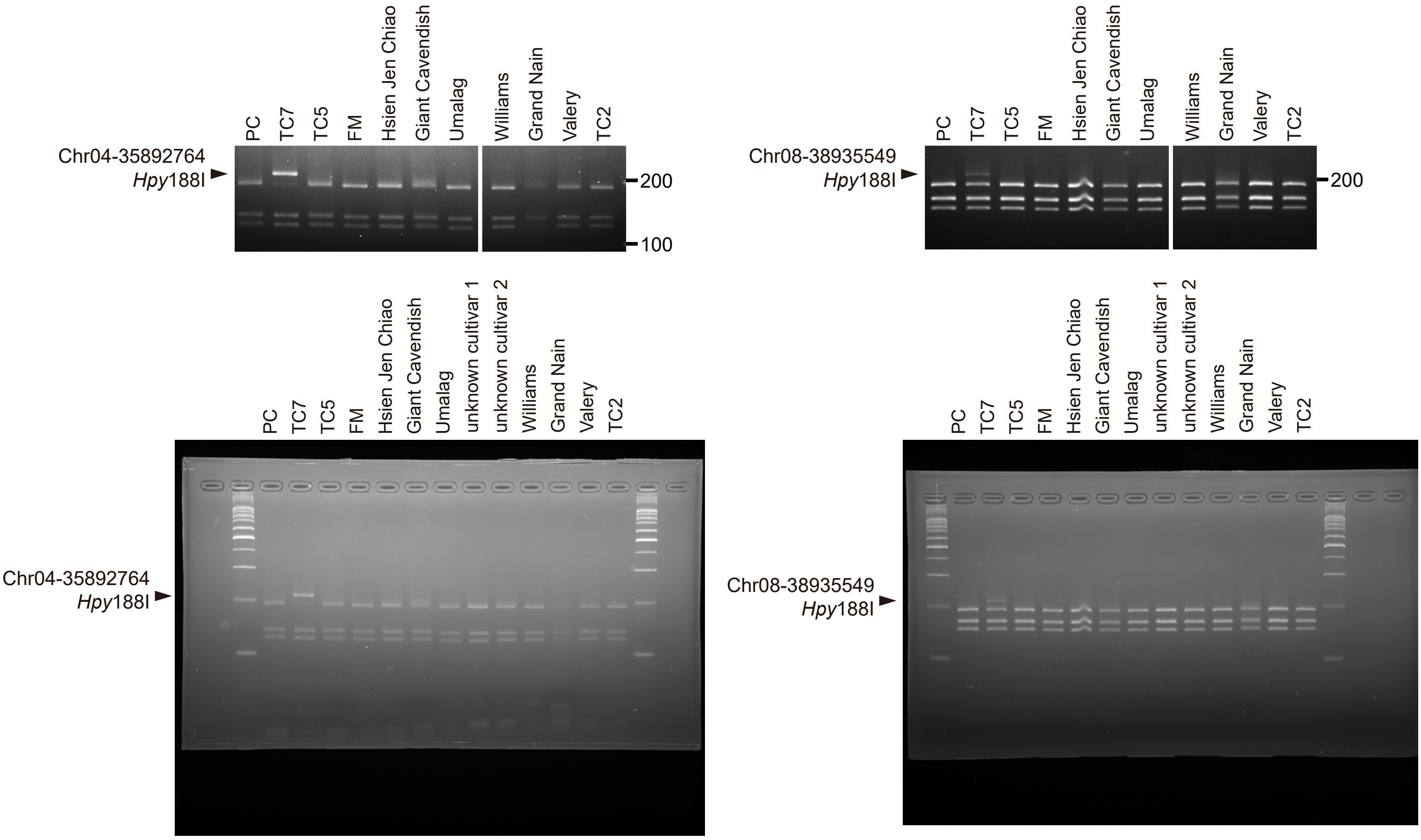

Figure S9. The uncropped full-length gels for TC7 CAPS/dCAPS marker in Figure 9B.

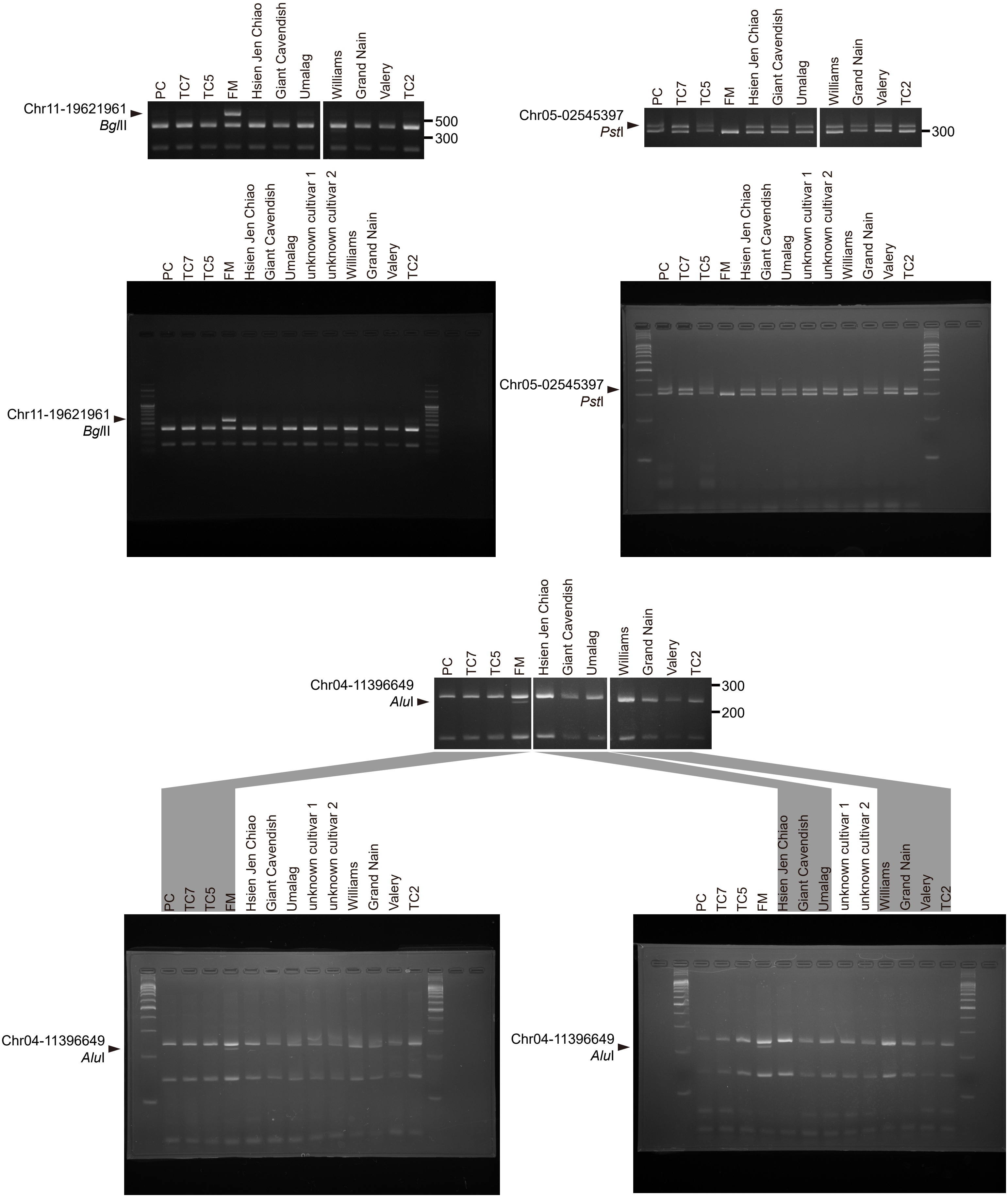

Figure S10. The uncropped full-length gels for FM CAPS/dCAPS marker in Figure 9C.

Table S1. Summary of the RNA-seq data used in this study.

SRA accession	ID (this study)	Cavendish cultivar	Repeat	Organ[a]	Foc TR4 susceptibility[b]	Batch[c]	Raw count	Mapped count	Length (nt)	Illumina platform	Reference
SRR12481170	PC-1	Pei-Chiao	1	ST	S	1 (2015/11)	37622308	27552032	126	HiSeq 2500	This study
SRR12481169	PC-2	Pei-Chiao	2	ST	S	1 (2015/11)	38692487	28224140	126	HiSeq 2500	This study
SRR12481168	PC-3	Pei-Chiao	3	ST	S	1 (2015/11)	33491445	23946422	126	HiSeq 2500	This study
SRR12481167	PC-4	Pei-Chiao	4	ST	S	2 (2016/10)	60106468	37792959	150	HiSeq 4000	This study
SRR16661002	TC2	Tai-Chiao No.2	1	ST	S	5 (2019/11)	24061168	16225880	150	NovaSeq 6000	This study
SRR12481166	TC5-1	Tai-Chiao No.5	1	ST	R	1 (2015/11)	39036101	28610841	126	HiSeq 2500	This study
SRR12481165	TC5-2	Tai-Chiao No.5	2	ST	R	1 (2015/11)	34317622	25158358	126	HiSeq 2500	This study
SRR12481164	TC5-3	Tai-Chiao No.5	3	ST	R	1 (2015/11)	35506273	26039165	126	HiSeq 2500	This study
SRR12481163	TC5-4	Tai-Chiao No.5	4	ST	R	2 (2016/10)	59327902	35489816	150	HiSeq 4000	This study
SRR12481162	TC7-1	Tai-Chiao No.7	1	ST	R	2 (2016/10)	67353186	42357368	150	HiSeq 4000	This study
SRR12481161	TC7-2	Tai-Chiao No.7	2	ST	R	3 (2017/07)	21589694	13618936	150	HiSeq 4000	This study
SRR15851055	FM-1	Formosana	1	ST	R	4 (2017/08)	29496010	19471232	150	HiSeq 4000	This study
SRR15851054	FM-2	Formosana	2	ST	R	4 (2017/08)	24978185	16145772	150	HiSeq 4000	This study
SRR15851053	FM-3	Formosana	3	ST	R	4 (2017/08)	25794384	16679019	150	HiSeq 4000	This study
SRR3406510	WL	Williams	1	MT	S	NA	26250721	8066107	90	HiSeq 2000	Gamez et al., BMC Genomics. 2019
SRR4188819	GN	Grand Nain	1	LF	S	NA	32971526	6721156	100	HiSeq 2000	Muthusamy et al., Front Plant Sci. 2016

[a] ST, shoots from tissue culture jars; MT, mixed tissues; LF, leaves.
[b] S, susceptible; R, resistant.
[c] Batch number in this study and the time (year/month) sequencing data received; NA, Not applicable.

Table S2. CAPS/dCAPS markers for TC5, TC7, and FM.

Marker	Chr	Position	Primer (5′ to 3′)[a]	PCR product size (bp)	Restriction enzyme	Digestion product size (bp)	
**TC5**						**TC5**	**others**
CAPS1	07	03224849	F: AGTATAGAGGCGGCAGAACAAG<br>R: CTCATTTTGCGTGCTCACTTAG	543	*Hpy*188I	455, 292, 163, 88	292, 163, 88
CAPS2	08	10837946	F: TGGTGGCAGAAATAGACTTGCT<br>R: CGACTCTTCCACCAGGCAAT	491	*Hind*III	491, 278, 213	278, 213
CAPS3	09	03683230	F: TCTTCTTTCCTTCGACAAAAGC<br>R: TTTGTAATGCCCTCCTTCATCT	623	*Pst*I	623, 441, 182	441, 182
**TC7**						**TC7**	**others**
dCAPS1	04	35892764	F: TCGCTTGCTTGGAGAAGTGTCAG<br>R: CTCCTCTTCCGACCTTACAGAC	508	*Hpy*188I	203, 181, 130, 118, 48, 22, 9	181, 130, 118, 48, 22, 9
dCAPS2	08	38935549	F: CGGTCAGCTAAATTTTGTTCTG<br>R: ATAAAGTGATCTCAATGTATCT	512	*Hpy*188I	207, 186, 162, 143, 21	186, 162, 143, 21
**FM**						**FM**	**others**
CAPS1	11	19621961	F: AAATGAGTACCTCGAAGTTGCA<br>R: AAAGCAATGATAACGTCAACCG	548	*Bgl*II	548, 391, 157	391, 157
dCAPS1	04	11396649	F: TGATTCCTTGACGTTCCCCT<br>R: CCGTGCTTCATTAGGTGAGC	371	*Alu*I	234, 215, 137, 19	234, 137
dCAPS2	05	02545397	F: CCCTCCTCTTTGTCGAAACCT<br>R: CTCTTATACCCGCCACAGGA	328	*Pst*I	304, 24	328, 304, 24

[a] The mismatched bases in the primer are underlined.

Table S3. List of 11 Cavendish banana cultivars used in the CAPS/dCAPS analysis and their countries of origin.

Cavendish cultivars	Foc TR4 susceptibility[a]	Country	Organ
Pei-Chiao (PC)	S	Taiwan	Mature leaves
Tai-Chiao No.7 (TC7)	R	Taiwan	Mature leaves
Tai-Chiao No.5 (TC5)	R	Taiwan	Mature leaves
Formosana (FM)	R	Taiwan	Mature leaves
Hsien Jen Chiao	S	Taiwan	Tissue culture seedlings
Giant Cavendish	S	Philippines	Tissue culture seedlings
Umalag	S	Philippines	Tissue culture seedlings
Williams	S	Hawaii	Tissue culture seedlings
Grand Nain	S	Honduras	Tissue culture seedlings
Valery	S	Honduras	Tissue culture seedlings
Tai-Chiao No.2 (TC2)	S	Taiwan	Tissue culture seedlings

[a] S, susceptible; R, resistant.

Table S4. Primer sequences used for the Sanger sequencing of the TC5, TC7, and FM SNVs.

	Chr	Position	Primer (5′ to 3′)	PCR product size (bp)
**TC5**				
	Chr04	36309122, 36309516, 36309562, 36309568, 36309614	F: TAGAAGATGAGCTGCAACGG<br>R: GATGTGCGCATGTGATGTTCGA	781
	Chr04	36316482	F: TGTGACTATGCATTGCTGATAAG<br>R: TGAGCTCATTGATTACTTGACCA	372
	Chr04	36322699, 36322712	F: TCACTGGAAAACTGCAACCG<br>R: AGCCTGCATCCATGGTTCTA	293
	Chr07	3224849	F: AGTATAGAGGCGGCAGAACAAG<br>R: CTCATTTTGCGTGCTCACTTAG	543
	Chr08	10837946	F: TGGTGGCAGAAATAGACTTGCT<br>R: CGACTCTTCCACCAGGCAAT	491
	Chr09	3683230	F: TCTTCTTTCCTTCGACAAAAGC<br>R: TTTGTAATGCCCTCCTTCATCT	623
	Chr11	25921279	F: ACCATCACCAGCTACGATGTCG<br>R: GGTGAGGTGATGATGGGCAGAT	346
	Chr11	26340252	F: AGGAAGCACCATACCAAGAGG<br>R: TCGTTTACGTTGTTCGTCAGC	187
**TC7**				
	Chr03	29971371	F: TCAGGTTCTTCACCTGTTACTGT<br>R: TGTAGCTGACAATGGTGGGCTT	372
	Chr04	96997	F: TCTCCTCTTGGCTTAGTTCTGCA<br>R: GGTGGAGGATGCTGTTGTCCTT	226
	Chr04	35204739	F: GCCCACAGAGCCACAAATTCAT<br>R: TCGGCGAGACATGAAAGCCTTA	288
	Chr04	35892764	F: GTCATCATCGAGGACTCCTGGG<br>R: ATTCGGTTCGAGCAATGCCTTG	249
	Chr05	887487, 887506, 887507	F: TGCTGATGATGGACGGAAGA<br>R: GCATCAATCAGGCTTTGTTCG	357
	Chr05	41281766, 41281829	F: CCGTGAGGAAGATGACGACT<br>R: TTTGTGATCACTCGGACCCC	428
	Chr07	9313503	F: TGCACAGGTGTTAATGCCTTGA<br>R: AGTTGAACAAACCTTCAGCGGC	220
	Chr08	38935549	F: AGATTACTGCTTTGCTGGTGGC<br>R: TGCACTTTACCATGCAGTTAGC	202
**FM**				
	Chr02	22586232	F: GTTGTTCTGAACTTGAGAGGCT<br>R: CTCCATCCTGCTGCAAAACTAA	475
	Chr03	6838117	F: TTTTGCTTTCCTGGTGCCTC<br>R: GCTGCTGTGTGGGGATTAGT	325
	Chr04	11396649	F: GGGCGGACTGAAGATCATTG<br>R: ACCCTTCCCAACTCCAATTCT	291
	Chr05	887507	F: TGCTGATGATGGACGGAAGA<br>R: GCATCAATCAGGCTTTGTTCG	357
	Chr05	2545397	F: TAGAGAACCGGTTACTGGCG<br>R: TCTGCCTCTCTATTCGTCCG	234
	Chr05	2661069	F: TGAGTACTATGGGCGATGCA<br>R: CTCGCCGCTTATTAAGTCAAAT	408
	Chr07	9385257	F: TAAAGTGGTAGCCCCTTTTCTG<br>R: CTGACCACGATACTCTTCTTCC	717
	Chr10	11138639	F: GACGCCCCTTTCATTGAGAC<br>R: GACACCGGGAAATCAAGCTC	467
	Chr11	19621961	F: AAATGAGTACCTCGAAGTTGCA<br>R: AAAGCAATGATAACGTCAACCG	548
